# Medication in older patients reviewed multiple ways (MORE) study

**DOI:** 10.1007/s11096-019-00879-3

**Published:** 2019-07-13

**Authors:** N. A. Zwietering, D. Westra, B. Winkens, H. Cremers, P. H. M. van der Kuy, K. P. Hurkens

**Affiliations:** 1 Department of Internal Medicine, Geriatric Medicine, Maastricht University Medical Centre, Maastricht University, PO Box 5800, 6202 AZ Maastricht, The Netherlands; 2 Department of Health Services Research, Care and Public Health Research Institute (CAPHRI), Maastricht University, Maastricht, The Netherlands; 3 Department of Methodology and Statistics, Care and Public Health Research Institute (CAPHRI), Maastricht University, Maastricht, The Netherlands; 4 Department of Clinical Pharmacy, Pharmacology and Toxicology, Zuyderland Medical Centre, Sittard-Geleen, The Netherlands; 5 Department of Hospital Pharmacy, Erasmus Medical Centre, Rotterdam, The Netherlands; 6 Department of Internal Medicine, Geriatric Medicine, Zuyderland Medical Centre, Sittard-Geleen, The Netherlands

**Keywords:** Clinical Decision Support System, Elderly, Medication review, Netherlands, Potentially inappropriate prescribing

## Abstract

*Background* Polypharmacy in older patients can lead to potentially inappropriate prescribing. The risk of the latter calls for effective medication review to ensure proper medication usage and safety. *Objective* Provide insight on the similarities and differences of medication review done in multiple ways that may lead to future possibilities to optimize medication review. *Setting* This study was conducted in Zuyderland Medical Centre, the second largest teaching hospital in the Netherlands. *Method* This descriptive study compares the quantity and content of remarks identified by medication review performed by a geriatrician, outpatient pharmacist, and Clinical Decision Support System. The content of remarks is categorized in seven categories of possible pharmacotherapeutic problems: ‘indication without medication’, ‘medication without indication’, ‘contra-indication/interaction/side-effect’, ‘dosage problem’, ‘double medication’, ‘incorrect medication’ and ‘therapeutic drug monitoring’. *Main outcome measure* Number and content of remarks on medication review. *Results* The Clinical Decision Support System (1.8 ± 0.8 vs. 0.9 ± 0.9, *p* < 0.001) and outpatient pharmacist (1.8 ± 0.8 vs. 0.9 ± 0.9, *p* = 0.045) both noted remarks in significantly more categories than the geriatricians. The Clinical Decision Support System provided more remarks on ‘double medication’, ‘dosage problem’ and ‘contraindication/interaction/side effects’ than the geriatrician (*p* < 0.050), while the geriatrician did on ‘medication without indication’ (*p* < 0.001). The Clinical Decision Support System noted significantly more remarks on ‘contraindication/interaction/side effects’ and ‘therapeutic drug monitoring’ than the outpatient pharmacist, whereas the outpatient pharmacist reported more on ‘indication without medication’ and ‘medication without indication’ than the Clinical Decision Support System (*p* ≤ 0.007). *Conclusion* Medication review performed by a geriatrician, outpatient pharmacist, and Clinical Decision Support System provides different insights and should be combined to create a more comprehensive report on medication profiles.

## Impacts on practice

- The Clinical Decision Support System is particularly potent in indicating ‘contraindications/interactions/side effects’ and can help reduce potentially inappropriate prescribing.
- Combined medication review by a geriatrician, an outpatient pharmacist, and a Clinical Decision Support System has different perspectives and should be combined to ensure adequate medication usage and safety. Moreover it could potentially reduce (increasing) health care costs.
- Benzodiazepines, betahistin, opiates, antihistamines, antihypertensive agents and non-steroidal-anti-inflammatory drugs provide for the majority of drug related problems in the Dutch hospital setting.

## Introduction

As the general population ages, the number of older patients (≥ 65 years) in hospitals increases [1–5]. These (ill) older patients make for a vulnerable group. This vulnerability is caused by factors such as reduced self-reliance due to multimorbidity, reduced cognitive skills, malnutrition, and physical constraints [6–8]. Because of their multimorbidity, polypharmacy (≥ 5 medicines) is common in this group. Besides their intended effects, these medicines have potential risks and adverse effects. The likelihood of the latter is significantly increased in older patients due to their vulnerability and altered pharmacokinetics and dynamics [9]. Prescribing medication in older patients is therefore complex and poses challenges.

The risk of potentially inappropriate prescribing (PIP) calls for effective methods to ensure adequate medication usage and safety. One of these methods is proper collaboration and coordination between patient or caregiver, physicians, pharmacists and other healthcare professionals. However, involvement of different physicians can cause a variety of medication prescriptions, increasing the risk of interactions and adverse effects if this is not taken into account whilst prescribing [10]. Geriatricians care for nearly the full spectrum of diseases seen in general medicine, taking into account the complexities in geriatric medicine. Therefore they often perform medication review: a structured evaluation of a patient’s medicines with the aim of optimising medicines use and improving health outcomes [11].

In the Netherlands medication review is mainly performed by geriatricians, pharmacists and general practitioners, using the STRIP (Systematic Tool to Reduce Inappropriate Prescribing) method. The national guideline focuses on analysing potential pharmacotherapeutic problems, including: undertreatment, ineffective medication, overtreatment, (potential) adverse effects, contraindication, interaction and dosage problem [12]. The STOPP/START (Screening Tool of Older Persons’ potentially inappropriate Prescriptions/Screening Tool to Alert Doctors to the Right Treatment) criteria [13, 14] are part of this analysis. The STOPP criteria describe PIP in older patients, whereas the START criteria provide guidance in prescribing medication in order to avoid undertreatment.

Although periodic medication review can lead to prevention of PIP [15, 16], there is no consensus on what such a review should comprise. In recent years, it has become apparent that Clinical Decision Support Systems (CDSSs) can provide support in medication reviews and could possibly reduce medication-related errors [17–19]. CDSSs could therefore contribute in improving quality of care and outcomes in older patients.

Moreover, pharmacists have extensive knowledge on pharmacokinetics and dynamics and could provide valuable insights in performing medication review. Hence, incorporating pharmacists in the process of medication review could improve prescribing appropriateness [20, 21].

## Aim of the study

In this observational study, the similarities and differences of medication reviews performed by a geriatrician, an outpatient pharmacist, and the hospital’s CDSS are described. We compare the quantity and types of possible pharmacotherapeutic problems identified by these three different perspectives. A better understanding of these perspectives could lead to possible optimization of medication review.

## Ethics approval

The study was approved by the Zuyderland Medical Ethical Testing Committee (METC) (study 16-N-115) and obtaining informed consent was waived.

## Method

### Setting

This study was conducted in Zuyderland Medical Centre, a large teaching hospital in the Netherlands that treats roughly 190,000 patients per year. The hospital is located in one of the fastest aging regions [22], hence many older patients are treated.

### Clinical rule reporter (CRR)

The CRR is a CDSS that is developed in the Zuyderland Medical Centre. It currently reviews medication profiles of all hospitalized patients. In addition, data on medication profiles of specific outpatient populations can be imported and reviewed. The system has access to all medication prescriptions of the past few months, through a digital network of regional pharmacies. Additionally, it has access to patient characteristics such as age, gender, co-medication and laboratory values. There is no digital link between the CRR and general practitioner data or medical history. The CRR combines information to obtain specific advice based on clinical rules (i.e. algorithms). These are clearly defined rules, that include the latest version of the STOPP/START criteria [13, 14] and utilize triggers to identify the need to discontinue or add medication, or aim for dose-reduction. During this study, 293 rules were operational, which is on top of medication surveillance for high-risk medication. The CRR reports by means of ‘green’ (no additional action needed) or ‘red’ (definite action needed) alerts. This implies the CRR checks for certain conditions by means of clinical rules and if this condition is met, a ‘green’ alert is noted [e.g. the use of proton pump inhibitors (PPIs) when using acetylsalicylic acid (ASA) at elder age]. On the other hand, hazardous combinations or an increased risk of adverse events, will generate a ‘red’ alert (e.g. the use of metformin in renal failure). Both ‘green’ and ‘red’ alerts are considered relevant because medication review by a physician or pharmacist should also comprise these checks. The clinical rules are updated based on insights from professional networks, research and guidelines.

### Data collection and analysis

Between May and July 2016, all new patients to the geriatric outpatient clinic were included (n = 200). There were no specific in- or exclusion criteria. Patient characteristics (i.e. gender, age, number of medications, and medication categorized to ATC (Anatomical Therapeutic Chemical Classification System) code [23]) were extracted from the electronic patient file and medication system. Based on the information available in the patient’s file, one of seven geriatricians performed a medication review (n = 200) on the day of visit using the national guideline [12]. The geriatricians were unaware of the study as medication review is considered part of the standard comprehensive geriatric assessment (CGA). The remarks on medication review were retrieved from the patient’s file by the research team. Furthermore, an outpatient pharmacist reviewed the patient’s medication independently in a consultation prior to the appointment at the geriatrician while having access to the same information as the geriatricians. These reviews were also guided by the previously mentioned national guideline. The remarks on medication review by the pharmacist were sent to the research team. Due to logistical challenges, 54 out of 200 medication profiles were reviewed by the pharmacist. These patients were selected randomly, based on presence of the pharmacist that day. Finally, 197 out of 200 medication profiles were assessed by the CDSS (3 patients did not consent to the digital exchange of data on medication use).

The remarks of the geriatrician, outpatient pharmacist and reports from the CDSS were categorized by the research team in seven pre-established categories (Table 1) that were used in previous research [24] and are based on possible pharmacotherapeutic problems [12]. Next, the quantity and content of remarks in these categories was compared. Moreover, the pharmacist focused on efficiency of medication prescriptions, drug adherence, use of over-the-counter (OTC) medications, and presence of a medication organizer or blister pack in addition to the seven categories. Drug adherence was assessed based on questions to patient or caregiver whether medication was used as prescribed and if there were any cognitive or practical barriers. It is important to specify that the geriatrician and outpatient pharmacist did not consult the CDSS for their patients. The information collected on medication review was shared in the patient’s file, accessible to all disciplines.

**Table 1:** Overview of remarks per patient by the geriatrician, outpatient pharmacist and CDSS

	Geriatrician (n = 200)	Outpatient pharmacist (n = 54)	CDSS (n = 197)
Total number of remarks	263	145	945
Characteristic (mean ± SD)			
Number of remarks	1.3 ± 1.5	2.7 ± 1.4	4.6 ± 3.6^b^
Number of different categories	0.9 ± 0.9	1.8 ± 0.8^a^	1.8 ± 0.8^b^
Categories (mean ± SD)			
Indication without medication	0.9 ± 1.0	1.3 ± 1.0^ac^	0.7 ± 1.0
Medication without indication	0.1 ± 0.6^b^	0.6 ± 0.8^ac^	0.0 ± 0.0
Contra-indication/interaction/adverse effect	0.0 ± 0.2	0.3 ± 0.5^a^	3.5 ± 2.7^bc^
Dosage problem	0.2 ± 0.5	0.4 ± 0.7^a^	0.3 ± 0.5^b^
Double medication	0.0 ± 0.0	0.0 ± 0.1	0.0 ± 0.0
Incorrect medication	0.0 ± 0.1	0.0 ± 0.0	0.0 ± 0.0
Therapeutic drug monitoring	0.0 ± 0.0	0.0 ± 0.0	0.1 ± 0.3^bc^

*SD* standard deviation

^a^Statistically significant for comparison between geriatrician and outpatient pharmacist

^b^Statistically significant for comparison between geriatrician and CDSS

^c^Statistically significant for comparison between outpatient pharmacist and CDSS

Statistical analysis was performed with IBM SPSS Statistics 23. Paired sampled *T* test was used to analyse differences in number and type of remarks between the geriatricians, outpatient pharmacist and CDSS. In these comparative analysis the corresponding sample size as mentioned above, was used. The Bonferroni correction was applied to address the problem of multiple comparisons. Continuous variables are reported as means (± standard deviation (SD)) and categorical variables as percentages.

## Results

In total 200 patients were included of which 118 (59%) were female. The mean age of the population was 82 (± 6) years. The mean number of medications used was 8 (± 4) including OTC and as required medications. Categorizing medication to ATC code, we see ≥ 40% of the patients use PPIs, vitamin D, anticoagulants, diuretics, beta blocking agents, agents acting on the renin–aldosterone–angiotensin system (RAAS), and statins (“Appendix 1”). More than 20% of the patients used drugs for constipation, mineral supplements (mainly calcium), cardiac therapy, analgesics other than opioids and hypnotics (mainly benzodiazepines).

The total number of remarks by the geriatrician was 263 (n = 200) compared with 145 (n = 54) by the outpatient pharmacist and 945 (n = 197) by the CDSS (809 ‘green’ and 136 ‘red’ reports). The mean number of remarks per patient by the geriatrician was 1.3 (± 1.5), compared with 2.7 (± 1.4) by the outpatient pharmacist and 4.6 (± 3.6) by the CDSS (*p* < 0.05). The outpatient pharmacist thus had twice as much remarks about the same patient than the geriatrician. In addition, the CDSS provides 1.8 times more remarks than the outpatient pharmacist and 3.6 times more than the geriatrician (both *p* < 0.001).

There are significant differences in the number of categories in which the geriatrician, outpatient pharmacist, and CDSS noted remarks. That is, the CDSS (1.8 ± 0.8 vs. 0.9 ± 0.9, *p* < 0.001) and outpatient pharmacist (1.8 ± 0.8 vs. 0.9 ± 0.9, *p* < 0.05) both noted remarks in more categories than the geriatricians. Regarding the specific categories, the CDSS provided more remarks on ‘double medication’, ‘dosage problem’ and ‘contraindication/interaction/adverse effects’ than the geriatrician did (*p* < 0.050), while the geriatrician provided more remarks on ‘medication without indication’ than the CDSS did (*p* < 0.001). The number of categories in which the outpatient pharmacist and CDSS noted remarks did not differ significantly (1.8 ± 0.8 vs. 1.8 ± 0.8, *p* = 0.458). However, the CDSS did note significantly more remarks in the ‘contraindication/interaction/adverse effects’ and ‘therapeutic drug monitoring’ category than the outpatient pharmacist, while the outpatient pharmacist noted more remarks than the CDSS in the ‘indication without medication’ and ‘medication without indication’ categories (*p* ≤ 0.007) (Table 1).

The three categories in which geriatricians reported remarks most commonly were ‘medication without indication’, ‘indication without medication’, and ‘contra-indication/interaction/adverse effect’. Analysis of the content of remarks in the ‘indication without medication’ category shows that mostly vitamins, analgesics, laxatives, and diuretics in patients with congestive heart failure were reported as missing medication. On the other hand benzodiazepines, betahistin, opiates and antihistamines frequently emerged as ‘medication without indication’. In the ‘contra-indication/interaction/adverse effect’ category, antihypertensive agents and non-steroidal-anti-inflammatory drugs (NSAIDs) were identified particularly as problem-causing medication.

The most commonly reported remarks by the outpatient pharmacist were on ‘indication without medication’, ‘medication without indication’ and ‘dosage adjustment’. Adequate pain medication, vitamin D, and calcium supplementation in case of insufficient dairy intake were advised in ‘indication without medication’. In contrast to the geriatricians, the largest share of remarks within the ‘medication without indication’ category concerns PPIs and a smaller share of betahistin and antihistamines. Finally, dosage adjustment is recommended especially for blood-glucose-lowering-agents and statins.

Besides remarks in the seven categories, the outpatient pharmacist focused on efficiency of medication prescriptions, drug adherence, use of OTC medications, and presence of a medication organizer or blister pack. These remarks are not included in Table 1. In this study, in 5.6% of the prescriptions there was a less expensive alternative. Drug adherence was assessed ‘well’ in 72.2% of the patients. Just over half (51.9%) of the patients indicated use of OTC medications (mostly vitamins and analgesics). A medication organizer or blister pack was present in 57.4% of the patients. The number of medications patients indicated to use, compared to the number in their medication profile, only corresponded in 29.6%.

Most of the CDSS reports are coded ‘green’ (85.6%), leaving the remainder ‘red’ (14.4%). This implies that most reports do not require prompt action of the physician, but in approximately one out of every seven patients, there are potential hazards. Most CDSS reports were on ‘contraindications/interactions/adverse effects’ with particular remarks on the use of opiates without laxatives, use of metformin in renal failure and use of tricyclic antidepressant combined with opioids or calcium antagonists. In “indication without medication”, the majority of reports was on use of PPIs in concomitant use of ASA.

**Fig. 1:**
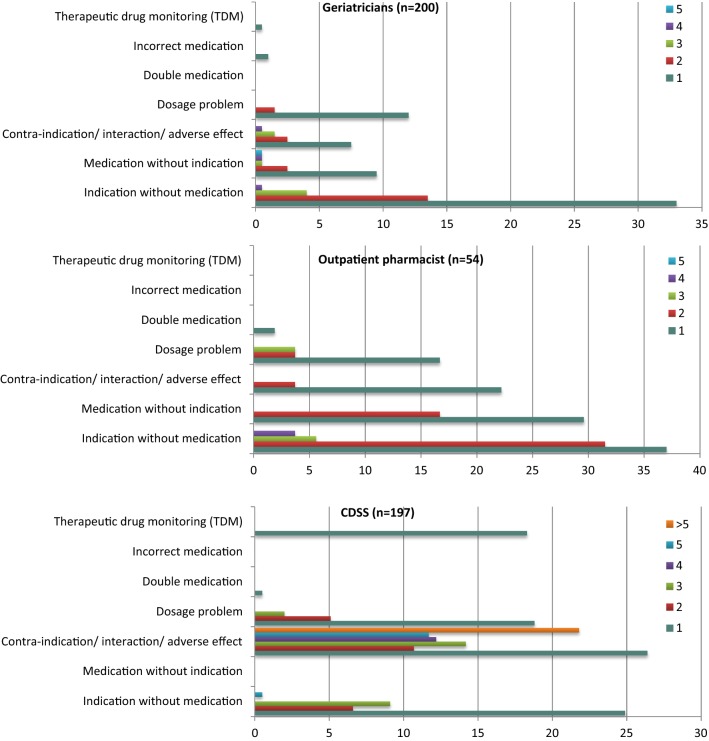
The number of remarks per category (% of patients)

Figure 1 shows in what percentage of patients a certain number of remarks was reported in one of the seven categories (also see “Appendix 2”).

## Discussion

### Different perspectives in medication review

This study showed that multiple ways of medication review lead to different perspectives. First, a lower total of remarks was noticed by the geriatrician, compared with the outpatient pharmacist and the CDSS. This probably has to do with the fact that the geriatrician was not specifically instructed to perform a medication review and the outpatient pharmacist was (instruction bias). In addition the geriatrician has limited time to take a patient’s history, perform a physical examination, interpret additional diagnostics and discuss a treatment plan. Limited time has previously been mentioned as one of the most important constraints in performing medication reviews [25]. It is questionable whether more reports by the outpatient pharmacist and more CDSS alerts lead to more clinically relevant reports. Despite the lower number of reports from the geriatricians, these all had clinical relevance as evaluated by an independent geriatrician. As a consequence of a standardized review guideline, there were no differences in the number of remarks between the geriatricians.

Moreover, the geriatricians mainly focused on ‘indication without medication’, ‘medication without indication’ and ‘contra-indication/interaction/adverse effect’, whilst the outpatient pharmacist focused on these first two and ‘dosage problem’. Regarding ‘indication without medication’ the similarities between the geriatrician and outpatient pharmacist include prescribing adequate pain-medication and vitamin D. The geriatrician focused on specific medical conditions such as congestive heart failure, whereas the outpatient pharmacist did on general problems such as insufficient dairy intake. The differences in nature and scope of the remarks could be explained by educational differences and clinical judgement on a different level by the geriatrician. Overall, all remarks in this category from both the geriatrician and outpatient pharmacist seemed clinically relevant.

Both the geriatricians and outpatient pharmacist considered betahistin and antihistamines as ‘medication without indication’. This is of particular interest because of the anticholinergic effects and potential adverse events associated with the use of antihistamines [26]. There was however a clear difference between both groups, as the outpatient pharmacist emphasizes that PPIs are used too often. Previous studies have shown that the use of PPIs amongst older patients is high and often inappropriate, and could cause potential harm [27]. Perhaps pharmacists are more aware of this problem, as adding a pharmacist to a medication review team more often causes de-prescribing of PPIs [28, 29]. However, the impact of de-prescribing PPIs on clinical outcomes is still not clear [29]. Lastly, the outpatient pharmacist had more remarks on ‘dosage problems’, possibly by a greater emphasis on and knowledge of pharmacokinetics.

### Pharmacist-provided medication review

In addition, the outpatient pharmacist provided information on efficiency of medication prescriptions, drug adherence, use of OTC medications, and presence of a medication organizer or blister. From an economic perspective, given the rising health-care costs in the Netherlands [30], it is interesting to discuss less expensive alternatives for medication. In this study, in 5.6% there was a less expensive alternative, suggesting that there already is emphasis on efficiency when prescribing medication, although it still provides possible cost reduction. Drug adherence was documented as ‘well’ in 72.2% of the patients, while just over half (57.4%) of the patients used a medication organizer or blister pack. Previous studies documented low drug adherence, particularly in chronic conditions, and adherence in such case is estimated to average 50% [31–33]. Despite the low number of reliable trials, some studies showed trends to improved adherence whilst using medication organizers or blister packs [34]. Adequate drug adherence is central to successful treatment of underlying diseases as non-adherence could lead to potential harm. This advocates focus on drug adherence during medication review and shows one of the benefits of including a pharmacist in this process. Overall, the pharmacist seems to have an important role in areas of medication safety, efficiency and drug adherence. Although previous literature showed neutral [35] and favourable [20, 36] evidence towards clinical and economical outcomes of pharmacist-provided medication review, we advocate pharmacist–geriatrician collaboration.

### Clinical Decision Support System

Focusing on the CDSS, there is particular potential in reviewing ‘contraindications/interactions/adverse effects’, as it is a system based on clinical rules. These rules, that include the STOPP/START criteria are useful in identifying PIP [37, 38]. Although most of the CDSS reports did not require prompt action from the physician, there were potential hazards in 14.4% of the medication profiles. The CDSS could therefore offer concrete and systematic ways of improving (de)prescribing medication and medication safety. Moreover, this set of clinical rules can be updated continuously and provides guidance to physicians working with older patients and polypharmacy. The disadvantage of CDSSs is the absence of clinical judgment and the fact that clinical rules apply at population level but not always on the individual.

### Joint medication review

Overall, there are pros and cons that arise from medication review performed by a geriatrician, outpatient pharmacist and CDSS. Because each reviewer has its qualities and disadvantages, an integrated multidisciplinary approach seems most desirable. Regardless of the way of medication review, prior research advocates interprofessional collaboration to achieve better clinical and economic outcomes [39]. One possible approach is using the CDSS in preparation of an outpatient visit and then have a (partly) joint geriatrician-pharmacist consultation. Of course, this would require time investment in preparation of consultations and less time for other clinical activities. On the other hand using the CDSS prior to consultations could possibly save time during medication review. Another approach is an outpatient clinic specialized in polypharmacy with joint geriatrician-pharmacist consultation, but this would only be feasible for a limited number of patients. Our future aim is a clear instrument for medication review with the least time investment and the greatest impact on clinical and economic outcomes. So far, to our knowledge, no big randomized trials have been conducted that focus on the effect of medication review in non-hospitalized older patients on long-term clinical and economic outcomes. The potentially beneficial effects of medication review or pharmacological intervention [40, 41] therefore require further research.

### Limitations

First of all, the outpatient pharmacist only reviewed a quarter of all medication profiles due to logistical challenges. Because patients were seen at different locations, it was not feasible for the outpatient pharmacist to be present at both. On the other hand, these patients were selected randomly and all statistical analyses were performed with paired T-tests in an attempt to correct this. Second, the geriatricians were not instructed specifically to perform a medication review and the outpatient pharmacist was. As a result, the number of remarks is not entirely comparable. Finally, this is a descriptive study, so no statements can be made on effects of medication review on clinical or economical outcomes.

## Conclusion

Medication review by geriatricians mainly focuses on clinically relevant problems for which medication should be initiated or discontinued. Evaluation by an outpatient pharmacist provides additional information on efficiency of prescriptions and drug adherence, that could reduce increasing health care costs and improve quality of care. The CDSS identified potential and therefore preventable hazards in 14% of the medication profiles. Because medication review performed by a geriatrician, outpatient pharmacist, and CDSS provides different insights it should be combined to create a more comprehensive report on medication profiles. However, further research is required to determine the effects on clinical and economic outcomes.

## Appendix 1

**Table:**

ATC code	Percentage of patients using medication (%)	ATC code	Percentage of patients using medication (%)	ATC code	Percentage of patients using medication (%)
A02A	1	B03B	12	M01	8
A02B	59	B03X	0.5	M04	6.5
A03	2	C01	23.5	M05	13.5
A04	1	C02	2.5	N02A	18.5
A05	0	C03	42	N02B	22
A06	23.5	C07	41	N03	6.5
A07	1	C08	18.5	N04	3.5
A10A	7.5	C09	48	N05A	4.5
A10B	18.5	C10	44	N05C	24.5
A11	42	D	10.5	N06A	18
A12	24.5	G	13.5	N06D	3
B01	64	H	13.5	R	17
B02	1	H02	6	S	13.5
B03A	3.5	J	2	OTHER	11.5

**Table:**

Definition of ATC codes
A02A antacids
A02B drugs for peptic ulcer and gastro-esophageal reflux disease (protonpumpinhibitors, H2 receptor antagonists)
A03 drugs for functional gastro-intestinal disorders
A04 anti-emetics and anti-nauseants
A05 bile and liver therapy
A06 drugs for constipation
A07 antidiarrheals, intestinal anti-inflammatory/anti-infective agents
A10A insulins and analogues
A10B blood glucose lowering drugs excl. insulins
A11 vitamins
A12 mineral supplements
B01 antithrombotic agents
B02 antihemorrhagics (e.g. vitamin K)
B03A iron preparations
B03B vitamin B12 and folic acid
B03X other anti-anemic preparations
C01 cardiac therapy
C02 anti-hypertensives
C03 diuretics
C07 beta blocking agents
C08 calcium channel blockers
C09 agents acting on the renin–angiotensin system
C10 lipid modifying agents
D dermatologicals
G genito-urinary system and sex hormones
H systemic hormonal preparations, excl. sex hormones and insulins
H02 corticosteroids for systemic use
J anti-infectives for systemic use
M01 anti-inflammatory and anti-rheumatic products
M04 anti-gout preparations
M05 drugs for treatment of bone disease
N02A opioids
N02B other analgesics and antipyretics
N03 anti-epileptics
N04 anti-Parkinson drugs
N05A antipsychotics
N05C hypnotics and sedatives
N06A antidepressants
N06D anti-dementia drugs
R respiratory system
S sensory organs

## Appendix 2

**Table:** Categories of remarks

No. of remarks	Category						
	Indication without medication	Medication without indication	Contra-indication/interaction/side-effect	Dosage problem	Double medication	Incorrect medication	Therapeutic drug monitoring (TDM)
Geriatrician (n = 200)							
0	98 (49%)	173 (86.5%)	176 (88%)	173 (86.5%)	200 (100%)	198 (99%)	199 (99.5%)
1	66 (33%)	19 (9.5%)	15 (7.5%)	24 (12%)	–	2 (1%)	1 (0.5%)
2	27 (13.5%)	5 (2.5%)	5 (2.5%)	3 (1.5%)	–	–	–
3	8(4%)	1 (0.5%)	3 (1.5%)	–	–	–	–
4	1 (0.5%)	1 (0.5%)	1 (0.5%)	–	–	–	–
5	–	1 (0.5%)	–	–	–	–	–
Outpatient pharmacist (n = 54)							
0	12 (22.2%)	29 (53.75%)	40 (74.1%)	41 (75.9%)	53 (98.1%)	54 (100%)	54 (100%)
1	20 (37%)	16 (29.6%)	12 (22.2%)	9 (16.7%)	1 (1.9%)	–	–
2	17 (31.5%)	9 (16.7%)	2 (3.7%)	2 (3.7%)	–	–	–
3	3 (5.6%)	–	–	2 (3.7%)	–	–	–
4	2 (3.7%)	–	–	–	–	–	–
5	–	–	–	–	–	–	–
CDSS (n = 197)							
0	116 (58.9%)	197 (100%)	6 (3%)	146 (74.1%)	196 (99.5%)	197 (100%)	161 (81.7%)
1	49 (24.9%)	–	52 (26.4%)	37 (18.8%)	1 (0.5%)	–	36 (18.3%)
2	13 (6.6%)	–	21 (10.7%)	10 (5.1%)	–	–	–
3	18 (9.1%)	–	28 (14.2%)	4 (2%)	–	–	–
4	–	–	24 (12.2%)	–	–	–	–
5	1 (0.5%)	–	23 (11.7%)	–	–	–	–
≥ 5	–	–	43 (21.8%)	–	–	–	–
